# Hierarchical dielectric orders in layered ferroelectrics Bi_2_SiO_5_

**DOI:** 10.1107/S2052252514008008

**Published:** 2014-04-30

**Authors:** Younghun Kim, Jungeun Kim, Akihiko Fujiwara, Hiroki Taniguchi, Sungwng Kim, Hiroshi Tanaka, Kunihisa Sugimoto, Kenichi Kato, Mitsuru Itoh, Hideo Hosono, Masaki Takata

**Affiliations:** a Department of Advanced Materials Science, The University of Tokyo, Kashiwa, Chiba 277-8561, Japan; b RIKEN SPring-8 Center, Kouto, Sayo-cho, Hyogo 679-5148, Japan; c Japan Synchrotron Radiation Research Institute, Kouto, Sayo-cho, Hyogo 679-5148, Japan; d Department of Physics, Nagoya University, Furo-cho, Nagoya 464-8602, Japan; e Department of Energy Science, Sungkyunkwan University, Jangan-Gu, Suwon 440-746, South Korea; f Department of Materials Science, Shimane University, 1060 Nishi-kawatsu-cho, Matsue, Shimane 690-8504, Japan; g Materials and Structures Laboratory, Tokyo Institute of Technology, Yokohama 226-8503, Japan; h Frontier Research Center, Tokyo Institute of Technology, Yokohama 226-8503, Japan

**Keywords:** electron charge density, electrostatic potential, visualization of local polarization, hierarchical dielectric ordering

## Abstract

Electric dipole engineering is now an emerging technology for high electron-mobility transistors, ferroelectric random access memory and multiferroic devices *etc*. Although various studies to provide insight into dipole moment behaviour, such as phase transition, order and disorder states, have been reported, macroscopic spontaneous polarization has been mainly discussed so far. Here, visualization of the electric dipole arrangement in layered ferroelectrics Bi_2_SiO_5_ by means of combined analysis of maximum entropy charge density and electrostatic potential distribution analysis based on synchrotron radiation X-ray powder diffraction data is reported. It was found that the hierarchical dipole orders, the weak-ferroelectric and ferroelectric configurations, were observed in the Bi_2_O_2_ and the SiO_3_ layers, respectively, and the ferrielectric configuration was realised by the interlayer interaction. This discovery provides a new method to visualize the local polarization in ferroelectric materials.

## Abstract 2

A hierarchical dielectric ordering in ferroelectric Bi_2_SiO_5_ was visualized by means of the maximum entropy method combined with electrostatic potential analysis *via* synchrotron radiation X-ray powder diffraction.

## 1. Introduction   

Designing and controlling the intense local electric field and/or polarization in solids is vital for emerging electronics, such as high-performance field-effect transistors, ferroelectric random access memory and multiferroic devices in the nano-scale (de Araujo *et al.*, 1995 ▶; Auciello *et al.*, 1998 ▶; Haertling, 1999 ▶; Scott, 2000 ▶; Dawber *et al.*, 2005 ▶; Schilling *et al.*, 2007 ▶; Chung *et al.*, 2011 ▶; Yamada *et al.*, 2012 ▶; Keeney *et al.*, 2012*a* ▶,*b* ▶, 2013 ▶; Maity *et al.*, 2012 ▶; Zhang *et al.*, 2012 ▶). Dielectric properties have been mainly discussed in terms of macroscopic properties based on measurements of dielectric permittivity (∊) and electric polarization (*P*) under electric fields (*E*) for bulk samples so far. Recently, electrostatic potential (EP) analysis based on electron charge density (ECD) using the maximum entropy method (MEM) has been developed for the characterization of specific features originating from the electrostatic field/force on the microscopic scale (Sakata & Sato, 1990 ▶; Takata & Sakata, 1996 ▶; Takata, 2008 ▶; Tanaka *et al.*, 2006 ▶; Kim *et al.*, 2011 ▶). Using ECD/EP analysis, we succeeded in visualizing the relationship between internal electric fields and physical properties, such as thermal conductivity affected by rattling (Fujiwara *et al.*, 2012 ▶) and superconductivity related to the bi-polaron (Kim *et al.*, 2014 ▶).

**Figure 1:**
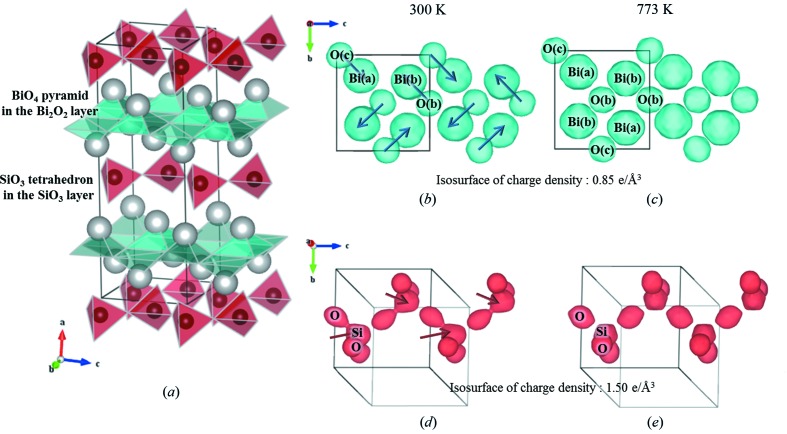
Schematic drawing of the crystal structure (*a*) and ECD using MEM distribution of the Bi_2_O_2_ (*b*, *c*) and the SiO_3_ layer (*d*, *e*) in the ferroelectric (300 K) and paraelectric (773 K) phases. The isosurface of ECD is 0.85 e Å^−3^ and 1.50 e Å^−3^ for the Bi_2_O_2_ and the SiO_3_ layer, respectively.

Bi_2_SiO_5_ (BSO) has attracted much attention as an alternative to the traditional lead-based ferroelectric materials with a phase-transition temperature (*T*_c_) of 673 K. BSO has an Aurivillius-like structure consisting of the [Bi_2_O_2_]^2+^ layer and [SiO_3_]^2−^ layer (Fig. 1*a* ▶) (Pirovano *et al.*, 2001 ▶; Georges *et al.*, 2006 ▶; Taniguchi *et al.*, 2013 ▶). A relatively large spontaneous polarization (*P*_*c*_) of 14.5 µC cm^−2^ along the SiO_3_ chain (*c*-axis) was predicted by first-principles calculations, while those along the *a*- and *b*-axes, *P*_*a*_ and *P*_*b*_, are estimated to be small, 0.1 µC cm^−2^ and 0 µC cm^−2^, respectively (Taniguchi *et al.*, 2013 ▶). From experimental *P* *versus* *E* measurements (Taniguchi *et al.*, 2013 ▶), only the *P*_*a*_ value of 0.8 µC cm^−2^ was detected, because the BSO crystals have a thin-plate shape, and the electrode for the *P* *versus* *E* measurements can only be formed on a large area of the *b*–*c* plane of the crystals. In addition, the polarization of BSO is suggested to originate from the SiO_3_ layer and not from the Bi_2_O_2_ layer by the first-principles calculations (Taniguchi *et al.*, 2013 ▶). Clarification of the origin and mechanism of the ferroelectricity in BSO is therefore crucial for further development of lead-free ferroelectric materials.

Here, we report the visualization of the electric dipole arrangement in layered ferroelectrics Bi_2_SiO_5_ by means of combined analysis of the ECD using MEM and EP distribution analysis based on high-precision synchrotron radiation X-ray powder diffraction data.

## 2. Experimental   

Synchrotron radiation X-ray powder diffraction measurements of BSO were performed at BL02B2 beamline at SPring-8 with a large Debye–Scherrer camera to obtain high counting statics for accurate structure analysis (Nishibori *et al.*, 2001 ▶; Takata *et al.*, 2002 ▶). The BSO sample was sufficiently ground for a homogeneous distribution of intensity and sealed in a glass capillary with a diameter of 0.1 mm. The diffraction pattern was measured at 300 K and 773 K with a N_2_ gas flowing temperature control system. The measurement wavelength was 0.35206 (1) Å to reduce absorption effects caused by the heavy atom (Bi) in the sample. The diffraction data were collected for 45 min on an image plate installed in the large Debye–Scherrer camera.

Determination of the precise structure was carried out by Rietveld refinement. Details of the process and the results are described in the supporting information. The total number of observed structure factors was 3921 and 2930 at 300 K and 773 K, respectively. The ECD was calculated by MEM using the *ENIGMA* program (Sakata *et al.*, 1990 ▶; Tanaka *et al.*, 2002 ▶). The electrostatic potential was calculated with a method developed by Tanaka *et al.* using the MEM electron charge density (Tanaka *et al.*, 2006 ▶). The electrostatic potential [*U*(*r*)] is composed of the nucleus charge [*U*_nuc_(*r*)] and the electron charge [*U*_ele_(*r*)] components. In this study, ECD and EP were visualized using the *OpenDx* program provided by IBM Visualization Data Explorer. The procedure for the polarization calculations is described in the supporting information.

## 3. Results   

**Figure 2:**
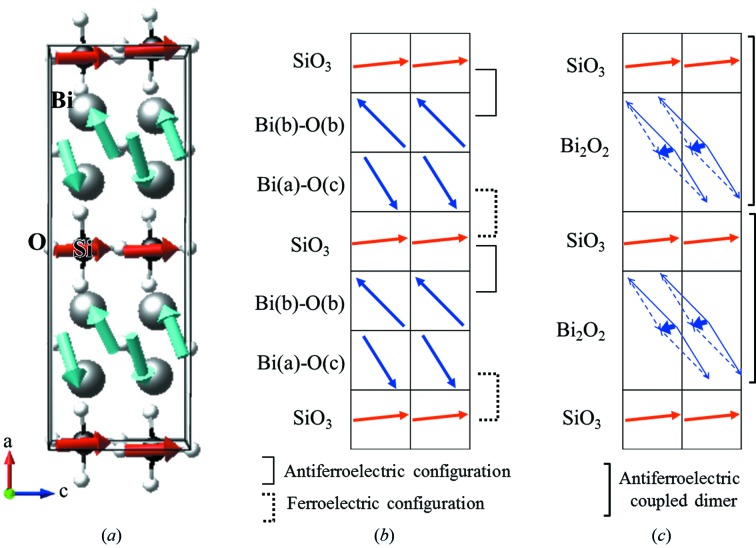
Schematic of the electric dipole configuration of Bi_2_SiO_5_ based on the polarization obtained by the ECD/EP method. (*a*) Three-dimensional electric dipole configuration with atom positions. (*b*) Schematics of dipole ordering for SiO_3_, Bi(*b*)–O(*b*) and Bi(*a*)–O(*c*). (*c*) Schematic dipole ordering showing a weak-ferroelectric configuration of the Bi_2_O_2_ layer. The residual dipole in the Bi_2_O_2_ layer is aligned antiparallel to the large dipole in the SiO_3_ layer showing the ferrielectric configuration.

The ECD/EP analysis is one of the best ways to understand the microscopic behaviour of polarizations in BSO. The ECD distributions directly obtained from integrated intensities of the X-ray diffraction pattern by MEM analysis reveal the deformation of both the BiO_4_ square pyramids in the Bi_2_O_2_ layer and the SiO_3_ tetrahedra in the SiO_3_ layer [Figs. 1(*b*)–1(*e*) ▶]. In the ferroelectric phase (300 K), the Bi atoms form a stronger covalent bond with one of the four equivalent first-neighbour O atoms in the paraelectric phase (773 K) (see Fig. S4 and Table S4 of the supporting information). The Bi(*b*)–O(*b*) and Bi(*a*)–O(*c*) pairs form electric dipole moments, and the two neighbouring electric dipoles form an almost antiparallel configuration in the Bi_2_O_2_ layer (Fig. 1*b* ▶). On the other hand, the Si atoms in the ferroelectric phase form a stronger covalent bond (Fig. 1*d* ▶) with three of the four equivalent first-neighbour O atoms in the paraelectric phase (Fig. 1*e* ▶), showing that the SiO_3_ cluster has an electric dipole moment. The electric dipoles of SiO_3_ align in the ferroelectric configuration. From the ECD analysis using MEM, the results visualized the antiferroelectric order in the Bi_2_O_2_ layer and the ferroelectric order in the SiO_3_ layer in the ferroelectric phase, as shown in Figs. 2(*a*) and 2(*b*) ▶. This is the reason why the large dipole moment of BSO originates from the SiO_3_ layer instead of the Bi_2_O_2_ layer.

**Figure 3:**
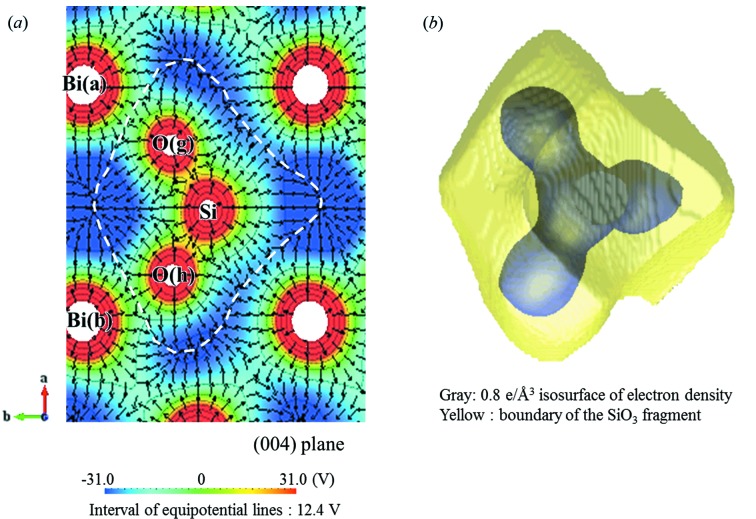
Extracted SiO_3_ fragment. (*a*) Two-dimensional EP map on the (004) plane with electric fields. The boundary defining the dipole unit can be determined by the local minimum value of the EP around the fragments (white dashed line). (*b*) Extracted three-dimensional perspective of the SiO_3_ fragment area (yellow) with the shape of the SiO_3_ molecule with an isosurface of 0.8 e Å^3^ (grey).

Electric dipole moments in the crystal can be calculated from the electron charge using MEM and the nuclear charge using Ewald’s method. It is, however, well known that the value of the polarization calculated from the charge distribution strongly depends on the method of selection of the crystallographic unit cell: the determination of the boundary of the crystallographic unit cell is critical for the calculation (Resta & Vanderbilt, 2007 ▶; Spaldin, 2012 ▶). This issue can be resolved by the Berry-phase theory (King-Smith & Vanderbilt, 1993 ▶; Resta, 1994 ▶; Neaton *et al.*, 2005 ▶). In the current case, we introduce the concept of fragments for extracting experimentally individual dipole units originating from BiO and SiO_3_ clusters. The boundary of fragments can be determined by the local minimum value of EP around the fragments (ECD/EP method) so that each fragment satisfies charge neutrality. An extracted fragment unit of SiO_3_ is shown in Fig. 3 ▶ as an example. The partial electric polarization in the fragments can be estimated by (Spaldin, 2012 ▶; Gohda *et al.*, 2000 ▶)
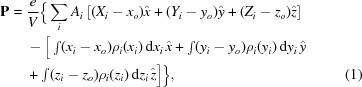
where *V* is the volume of the unit cell; *e* is the elementary charge (1.602 × 10^−19^ C); *A*_*i*_ is atomic number; (*X*_*i*_, *Y*_*i*_, *Z*_*i*_) is the position of *i*th atom; (*x*_*o*_, *y*_*o*_, *z*_*o*_) is the position of the center of mass in the fragment unit; ρ_*i*_(*x*_*i*_, *y*_*i*_, *z*_*i*_) is the electron density located at the *i*th pixel; (*x*_*i*_, *y*_*i*_, *z*_*i*_) is the position of *i*th pixel for electron charge contribution; 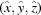 is the unit vector. Integration is carried out over the fragment unit; the value of the electron density is assigned to pixels in a unit cell divided into 256 × 128 × 128 pixels. The total and projected values of polarization are summarized in Table 1 ▶.

The SiO_3_ layer shows a large polarization along the *c*-axis originating from a large dipole moment of the SiO_3_ fragment [27.3 (1) µC cm^−2^]. The projected values of the polarization along the *a*- and *c*-axis, *P*_*a*_ and *P*_*c*_, in the SiO_3_ layer are 1.4 (1) µC cm^−2^ and 27.3 (1) µC cm^−2^, respectively. It should be noted that *P*_*b*_ is zero due to the inversion symmetry operation along the *b*-axis. On the other hand, the Bi_2_O_2_ layer has a small but distinct polarization value in spite of the antiferroelectric order: the projections of the polarization in the Bi_2_O_2_ layers were −1.8 (1) µC cm^−2^ for *P*_*a*_ and −3.8 (1) µC cm^−2^ for *P*_*c*_. This originates from the asymmetric distortion of the Bi_2_O_2_ pyramids, meaning that the Bi–O dipoles with antiparallel configuration do not fully cancel out the polarization in the layer: this is regarded as the weak-ferroelectric configuration, as shown in Fig. 2(*c*) ▶. Since the residual polarization in the Bi_2_O_2_ layer aligns in the antiparallel direction to the polarization in the SiO_3_ layer, the distortion in the Bi_2_O_2_ layer is suggested to be induced by the large polarization of the SiO_3_ layer for reducing the electrostatic energy in the crystal. The individual dipole moment [26.8 (3) µC cm^−2^ and 27.0 (4) µC cm^−2^] of Bi–O is comparably large with that of the SiO_3_ fragment [27.3 (1) µC cm^−2^], although the net value is as small as 4.2 (1) µC cm^−2^ owing to the antiparallel configuration of the Bi–O dipoles.

The total *P*_*a*_ and *P*_*c*_ of BSO were estimated to be 0.3 (2) µC cm^−2^ and 23.5 (1) µC cm^−2^, respectively. *P*_*b*_ was zero because of the symmetry operation of the crystal structure. The value of *P*_*a*_ is roughly consistent with that predicted by theoretical calculation (0.1 µC cm^−2^) and that determined by *P* *versus* *E* measurements (0.8 µC cm^−2^) (Taniguchi *et al.*, 2013 ▶). In addition, the large *P*_*c*_ value predicted by theoretical calculation [14.5 µC cm^−2^] was experimentally determined by a microscopic approach using ECD/EP analysis [23.5 (1) µC cm^−2^]. The result shows that the ECD/EP analysis using precise X-ray diffraction data can derive the local electric dipole moment in the crystal as well as in the polarization values from small amounts (less than 0.1 mg) of powder samples, and values are consistent with those predicted by the complete picture based on the Berry-phase theory. It should be noted that the values of polarization based on the point charge model, where the electron charge of atoms was assigned to each atomic position obtained by Rietveld analysis, largely deviated from any other results of theoretical prediction, *P* *versus* *E* measurements and the ECD/EP analysis; this result shows that the use of the ECD distribution is essential for estimation of accurate values of polarization. The method of ECD/EP analysis is, therefore, useful for characterization and design of newly synthesized dielectric materials, and thus for the development of emerging dielectric materials.

The hierarchical dipole ordering with structural distortion can be understood in terms of electrostatic energy. Firstly, the antiferroelectric configuration with the residual dipole moments (weak ferroelectricity) and the ferroelectric configuration are realised in the Bi_2_O_2_ and the SiO_3_ layer, respectively. Next, the antiparallel arrangement between the net small dipoles in the Bi_2_O_2_ layer and the large dipoles in the SiO_3_ layers reduces the interlayer electrostatic interaction; this configuration is regarded as an interlayer ferrielectric ordering. In addition, the neighbouring Bi_2_O_2_ and SiO_3_ layers form a pair, namely, the dimerization of the layers (Figs. 2*c* ▶ and S3*a*). In this distortion the dipoles in the neighbouring layer being aligned in the antiparallel configuration become close to reduce the electrostatic energy. In actual fact, the cohesive energy of the low-temperature ‘ferroelectric’ phase is lower than that of the high-temperature paraelectric phase by 23.1 meV in Bi_2_SiO_5_ (Taniguchi *et al.*, 2013 ▶), showing an energetic advantage of the ‘ferroelectric’ phase with hierarchical dipole ordering.

The ECD/EP analysis can visualize the properties of covalency and polarization in the crystal using a tiny amount of powder sample. In addition, the method demonstrates its ability to visualize local polarization which cannot be detected by the macroscopic measurements in principle. In the case of BSO, for instance, a large dipole moment should be induced at the SiO_3_ layer, and large polarization is stabilized by the antiparallel configuration with the Bi_2_O_2_ layer.

## 4. Summary   

In summary, we have discovered a new approach to the visualization of the local electric dipole moments and their orders in a crystal by means of the combined analysis of ECD using MEM and EP distribution analysis based on synchrotron radiation X-ray powder diffraction data. Application of this method revealed hierarchical dipole ordering in Bi_2_SiO_5_: the weak ferroelectricity in the Bi_2_O_2_ layer, the ferroelectric order in the SiO_3_ layer, and the ferrielectric order between the Bi_2_O_2_ and SiO_3_ layers. The results suggest that ECD/EP analysis is a useful method to visualize the local polarization based on X-ray powder diffraction experiment.

## Supplementary Material

Determination of the local and the total polarization values and precise structural parameters; covalency revealed by MEM charge density distribution analysis; fragments determined by electrostatic potential boundary. DOI: 10.1107/S2052252514008008/ro5001sup1.pdf

## Figures and Tables

**Table 1:** Total and projected polarization estimated by the ECD/EP method, point charge model (PC model), *PversusE* measurement and first-principles calculation *PversusE* measurements and the first-principles calculation are reported by Taniguchi *et al.* (2013 ▶). All values are shown in units of Ccm^2^.

		Total polarization	Projected polarization		
		|*P*|	*P*_*a*_	*P*_*b*_	*P*_*c*_
ECD/EP method	Bi_2_O_2_ layer	4.2(1)	1.8(1)	0	3.8(1)
	Bi(*b*)O(*b*) sublayer (upper square-pyramid component)	26.8(3)	24.0(3)	0	11.9(1)
	Bi(*a*)O(*c*) sublayer (lower square-pyramid component)	27.0(4)	25.8(4)	0	8.0(1)
	SiO_3_ layer	27.3(1)	1.4(1)	0	27.3(1)
	Bi_2_SiO_5_	23.5(1)	0.3(2)	0	23.5(1)
PC model	Bi_2_SiO_5_	9.4	4.9	0	8.1
*P versus E* measurement	Bi_2_SiO_5_		0.8		
First-principles calculation	Bi_2_SiO_5_	14.5	0.1	0	14.5
