# Supplementary material for: Hierarchical dielectric orders in layered ferroelectrics Bi2SiO5
Source: IUCrJ. 2014 Apr 30;1(Pt 3):160–4. doi: 10.1107/S2052252514008008 (PMC4086432; doi:10.1107/S2052252514008008)
Supplement: Supplementary file 1 [file m-01-00160-sup1.pdf]

IUCrJ

Volume 1 (2014)

Supporting information for article:

Hierarchical dielectric orders in layered ferroelectrics Bi2SiO5

Younghun Kim, Jungeun Kim, Akihiko Fujiwara, Hiroki Taniguchi, Sungwng Kim, Hiroshi Tanaka, Kunihisa Sugimoto, Kenichi Kato, Mitsuru Itoh, Hideo Hosono and Masaki Takata

S1. Procedure of analysis for the determination of the local and the total polarization values.

A flowchart of the polarization analysis is shown in Figure S1. Precise synchrotron radiation (SR) X-ray powder diffraction (XRD) data of Bi_2SiO_5 (BSO) at 300 K (ferroelectric phase) and 773 K (paraelectric phase) were analyzed by the MEM/Rietveld method (Sakata & Sato, 1990; Takata & Sakata, 1996; Takata, 2008; Tanaka *et al.*, 2006; Kim *et al.*, 2011). Maximum Entropy Method (MEM) was iteratively performed until the condition of C=1 is satisfied in the following equation (Sakata & Sato, 1990):

$$C = \frac{1}{N}\sum \frac{\left(F(k)_{obs} - F(k)_{MEM}\right)^2}{\sigma(k)^2}$$

where, N is the number of structure factors, $F(k)_{obs}$ is the observed structure factor from the experimental measurement, $\sigma(k)$ is the standard deviation of observed structure factor, $F(k)_{MEM}$ is the calculated structure factor from MEM charge density. For Rietveld refinement, the iteration was continued until the value of estimated standard deviation is less than 5 %.

In the conventional point charge (PC) model, polarization values were calculated using atomic positions obtained by Rietveld refinement (the result of the final cycle of Rietveld analysis) as the positions of charge, with value of valence electrons for each atom. In the present work, we carried out electron charge density and electrostatic potential (ECD/EP) analysis (Tanaka *et al.*, 2006; Kim *et al.*, 2011; Fujiwara *et al.*, 2012; Kim *et al.*, 2013) for the evaluation of the polarization values. ECD distribution obtained by the final cycle of MEM analysis was used as the positions of negative charge, while atom positions by Rietveld analysis were used as for nuclear charge. For the extraction of the unit of dipole in the ECD/EP analysis, the trace of local minimum position of EP was defined as a boundary of the unit: the spatial variation of EP, $U(r)$, is obtained by the sum of electron- and nucleus-charge components, $U_{\mathrm{ele}}(r)$ and $U_{\mathrm{nuc}}(r)$, respectively. The $U_{\mathrm{ele}}(r)$ was directly calculated from the MEM charge density in the reciprocal space. On the other hand, the $U_{\mathrm{nuc}}(r)$ is estimated by the ordinary Ewald's method using atomic positions, taking into account anisotropic thermal displacement estimated by the complementary MEM/Rietveld anlaysis (Kim *et al.*, 2009).

The partial electric polarization in the ECD/EP analysis is obtained by the equation (1) in the main text. The electron density inside the fragment determined by the EP boundary was quantized, because of the finite size of the pixel in the unit cell (divided into 256×128×128 pixels). The error due to this method was estimated by increasing or decreasing 1 pixel from the position of the local minimum EP boundary.

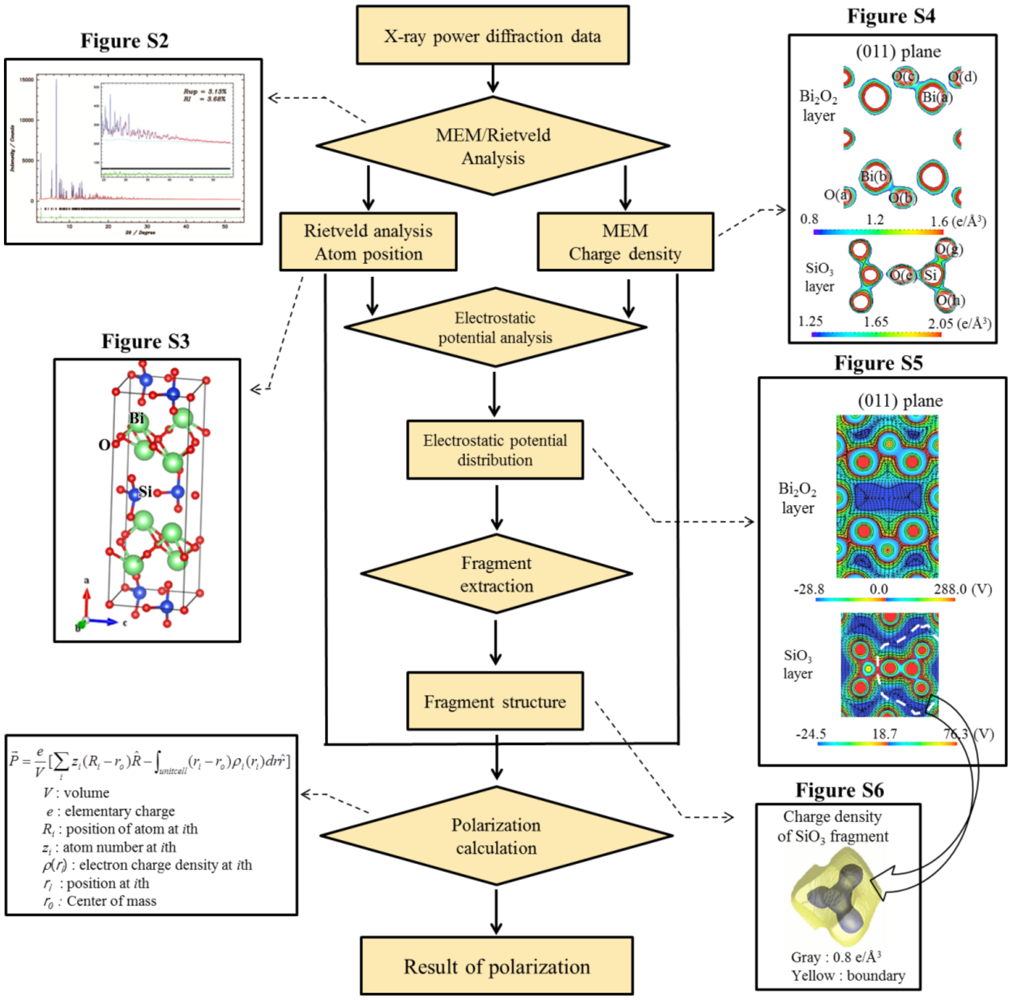

Figure S1 Flowchart for the determination of the local and the total polarization value.

S2. Precise structural parameters determined by Rietveld analysis.

SR XRD data with the profiles based on results of Rietveld refinement are shown in Figure S2, and the obtained structural parameters, atomic positions, and temperature displacement parameters are summarized in Tables S1-S3. Reliability of Rietveld analysis was evaluated by the *R*-factors (R_{wp} : weighted profile reliability factor, R_I : integrated intensity reliability factor) (Brian, 2006),

$$R_{\mathrm{wp}} = \left[\frac{\sum_i w_i (y_i - y_{i,calc})^2}{\sum_i w_i y_i^2} \right]^{\frac{1}{2}}, \quad R_I = \frac{\sum_{hkl} \left| I_{hkl} - I_{hkl,calc} \right|}{\sum_{hkl} I_{hkl}},$$

where w_i is the weight, y_i is the observed intensity at *i*th, $I_{hkl} = mF^2_{hkl}$ (m is the multiplicity, F_{hkl} is the structure factor). Low values of the *R*-factors (< 4 %) assure the structural parameters determined by Rietveld refinements. Structures in both phases of ferroelectric and paraelectric states show a layered structure which is composed of the SiO_3 and the Bi_2O_2 layers. BSO has a structural phase transition from orthorhombic (*Cmcm*) to monoclinic (*Cc*) structure with subtle change of β angle as temperature decreases. In the ferroelectric phase at 300 K, the SiO_3 layer is slightly moved to the $[\bar{1}00]$ direction (Figure S3a). It causes unequal interlayer distance to the adjacent Bi_2O_2 layers: forming the dimerization of the SiO_3 and the Bi_2O_2 layers.

(a)

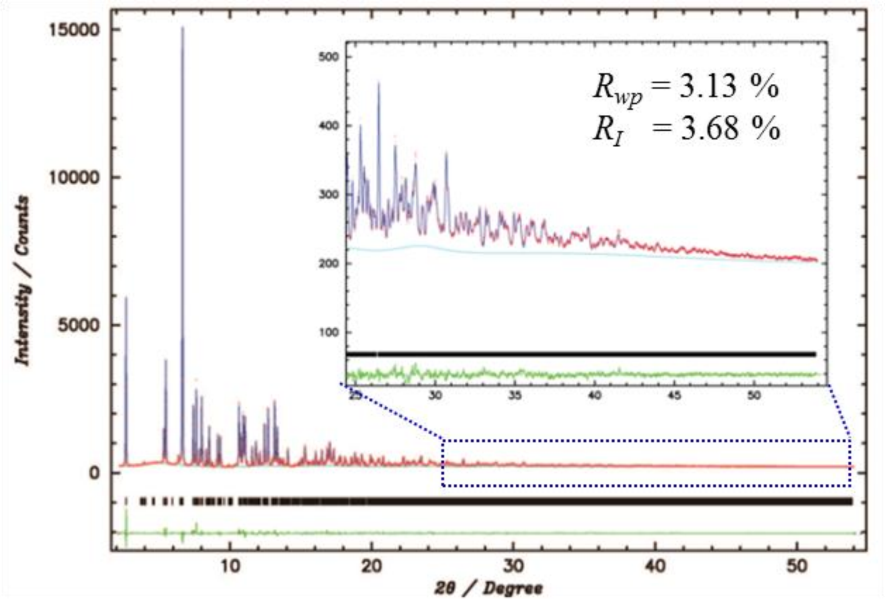

(b)

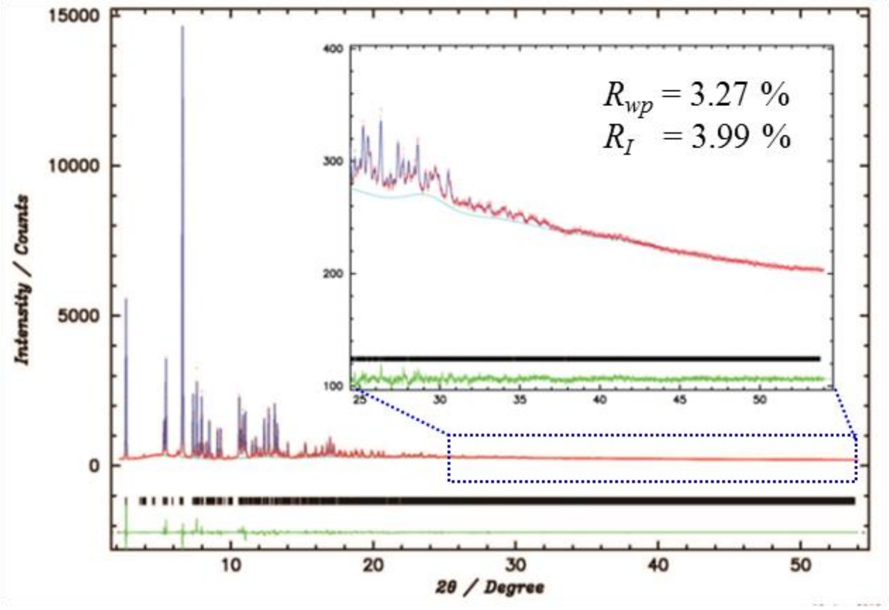

Figure S2 Results of Rietvled refinement at (a) 300 K and (b) 773 K. *R*-factors are shown in the figure. The observed and calculated data are indicated by the red dots and the blue line. The sky blue line shows background. The green line shows the difference between the calculated and observed data.

	300 K (Ferroelectric phase)	773 K (Paraelectric Phase)
Space group	*Cc* (#9)	*Cmcm* (#63)
a (Å)	15.1193(1)	15.1968(1)
b (Å)	5.4435(1)	5.4843(1)
c (Å)	5.2892(1)	5.2964(1)
β (degree)	90.070(2)	90
Volume (Å^3)	435.31(2)	441.42(2)
R_{wp} (%)	3.13	3.27
R_I (%)	3.68	3.99

Table S1 Space group, lattice parameters, lattice volume and *R*-factors obtained by Rietveld analysis at 300 K and 773 K. BSO has structural phase transition from orthorhombic (*Cmcm*) to monoclinic (*Cc*) structure with subtle change of β angle.

(a) 300 K

Atom	Site	*x*	*y*	*z*
Bi(a)	4a	0.170(1)	0.217(1)	0.261(1)
Bi(b)	4a	0.840(1)	0.219(2)	0.274(5)
Si	4a	0.496(1)	0.172(1)	0.246(1)
O(a),O(b)	4a	0.250(2)	0.494(9)	0.495(6)
O(c),O(d)	4a	0.256(2)	0.007(12)	0.035(10)
O(e),O(f)	4a	0.490(2)	0.017(2)	0.511(5)
O(g)	4a	0.586(1)	0.331(2)	0.225(4)
O(h)	4a	0.404(1)	0.323(2)	0.227(4)

(b) 773 K

Atom	Site	*x*	*y*	*z*
Bi	8g	0.165(1)	0.218(1)	0.250
Si	4c	0	0.686(1)	0.250
O(a~d)	8e	0.248(1)	0.500	0.500
O(e),O(f)	4b	0	0.500	0.500
O(g),O(h)	8g	0.092(1)	0.181(2)	0.750

Table S2 Atom position of BSO for (a) monoclinic phase at 300 K and (b) orthorhombic phase at 773 K.

(a) 300 K

Atom	U	U_{11}	U_{22}	U_{33}
Bi(a)	-	0.008(1)	0.008(1)	0.007(1)
Bi(b)	-	0.008(1)	0.008(1)	0.007(1)
Si	-	0.013(3)	0.010(3)	0.012(4)
O(a),O(b)	0.029(5)	-	-	-
O(c),O(d)	0.029(5)	-	-	-
O(e),O(f)	-	0.022(9)	0.031(9)	0.031(9)
O(g),O(h)	0.029(5)	-	-	-

(b) 773 K

Atom	U	U_{11}	U_{22}	U_{33}
Bi	-	0.024(1)	0.022(1)	0.025(1)
Si	-	0.019(4)	0.022(4)	0.016(4)
O(a~d)	0.039(3)	-	-	-
O(e),O(f)	-	0.033(11)	0.040(10)	0.049(13)
O(g),O(h)	0.039(3)	-	-	-

Table S3 **Thermal displacement parameters of BSO at (a) 300 K and (b) 773 K.** Thermal displacement parameters are determined by the shape of electron density at each atom. U is the isothermal displacement parameter. U_{11}, U_{22} and U_{33} are the thermal displacement of x, y and z direction, respectively.

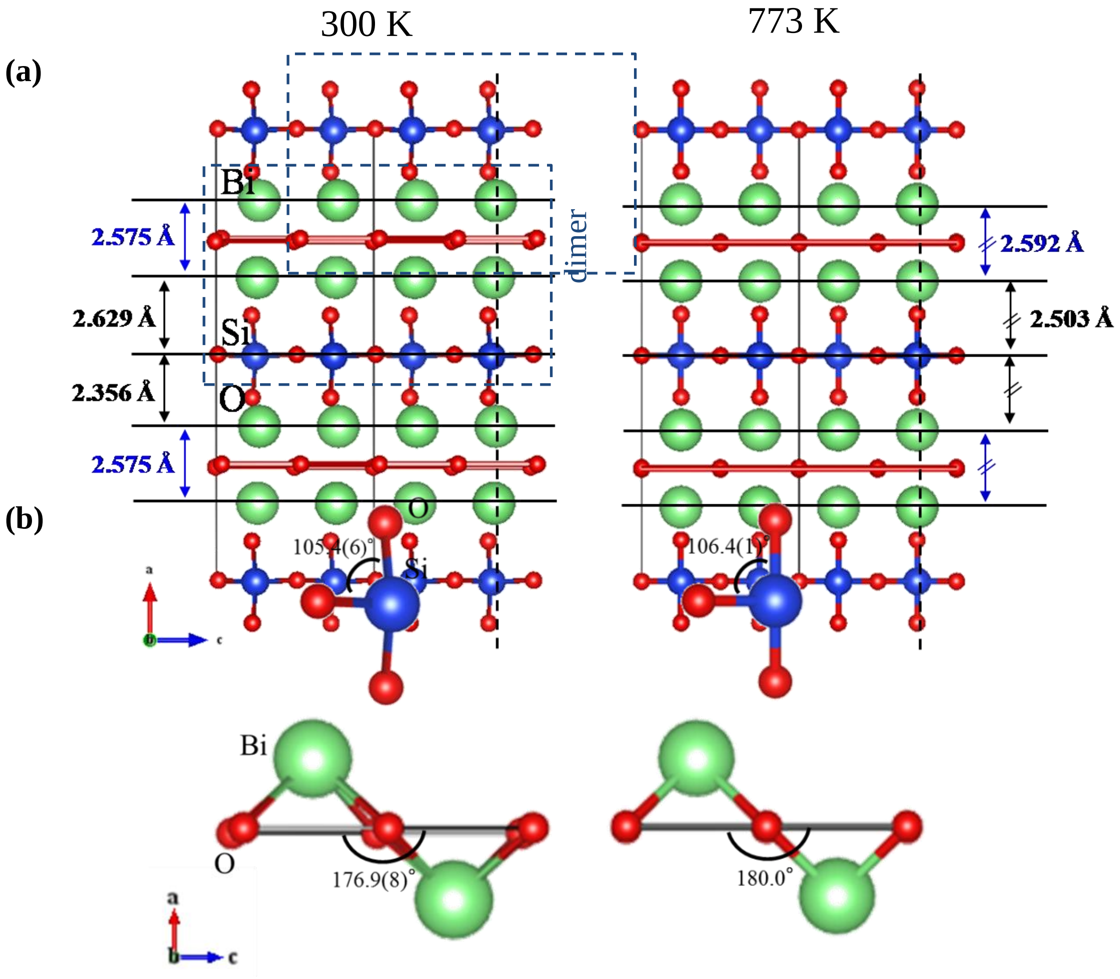

Figure S3 **Distorted local structure of ionized layer at ferroelectric phase. (a)** Crystal structure of BSO projected onto the out-of-plane at each temperature region. The pair of SiO_3 and Bi_2O_2 layers forms a "dimer" due to the displacement of the SiO_3 layer and the Bi_2O_2 in the $[\bar{1}00]$ direction **(b)**.

S3. Covalency revealed by MEM charge density distribution analysis

The results of MEM charge density of BSO (2-dimensional contour map of the (011) plane) are shown in Figure S4. Reliability of MEM analysis was evaluated by the R_{MEM}-factor (Sakata & Sato, 1990),

$$R_{\mathrm{MEM}} = \frac{\sum \left| F(k)_{obs} - F(k)_{MEM} \right|}{\sum \left| F(k)_{obs} \right|},$$

where $F(k)_{obs}$ and $F(k)_{MEM}$ are structure factor obtained from structural refinement and MEM electron charge density, respectively. The values of R_{MEM} were 3.72 % and 4.11 % for ferroelectric (300 K) and paraelectric (773 K) phases, respectively. The details of bonding electron density between cation and anion are listed in Table S4. The bonding electron density of Bi(b)-O(b) and Bi(a)-O(c) is significantly enhanced in the Bi_2O_2 layer (Figure S4, Table S4). In addition, the electron density distribution around Si ion was changed into an asymmetrical shape. The formation of fragments which has an electric dipole moment was observed; it is strongly associated to the ferroelectricity of BSO.

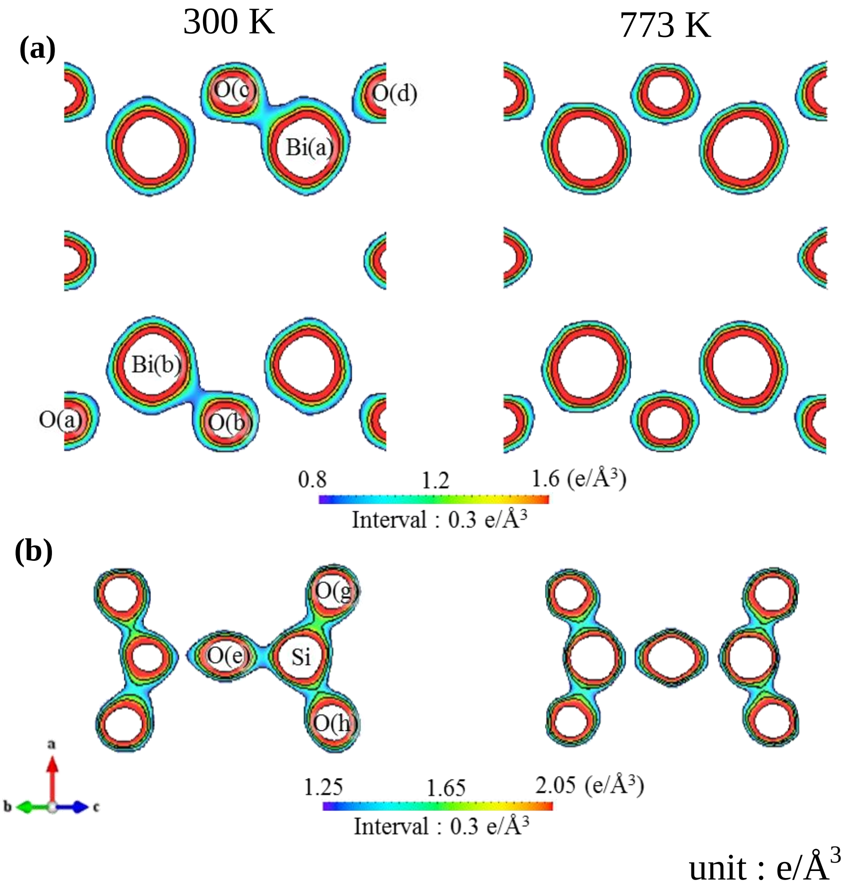

Figure S4 Two-dimensional charge density map of (a) the Bi_2O_2 and (b) the SiO_3 layer.

Cation	Anion	300 K	773 K
Bi(a)	O(a)	0.65(1)	0.65(2)
	O(b)	0.74(2)	0.65(2)
	O(c)	0.98(1)	0.65(1)
	O(d)	0.71(1)	0.65(1)
Bi(b)	O(a)	0.68(1)	0.65(1)
	O(b)	0.85(1)	0.65(1)
	O(c)	0.68(1)	0.65(2)
	O(d)	0.69(1)	0.65(2)
Si	O(e)	1.57(1)	1.29(8)
	O(f)	1.42(1)	1.29(8)
	O(g)	1.61(1)	1.54(3)
	O(h)	1.54(2)	1.54(3)

Table S4 Values of bonding electron charge density between cation and anion.

S4. Fragments determined by electrostatic potential boundary.

Figure S5 shows two-dimensional electrostatic potential (EP) maps of the (011) plane. The dipole produced by the formation of a pair between Bi(Si) and O can be also confirmed by the EP distribution. The determined fragment by EP boundary, which is the trace of local minimum position of EP, is shown as the white dashed line in the Figure S5. Extracted three-dimensional perspective of fragments is shown in the Figure S6.

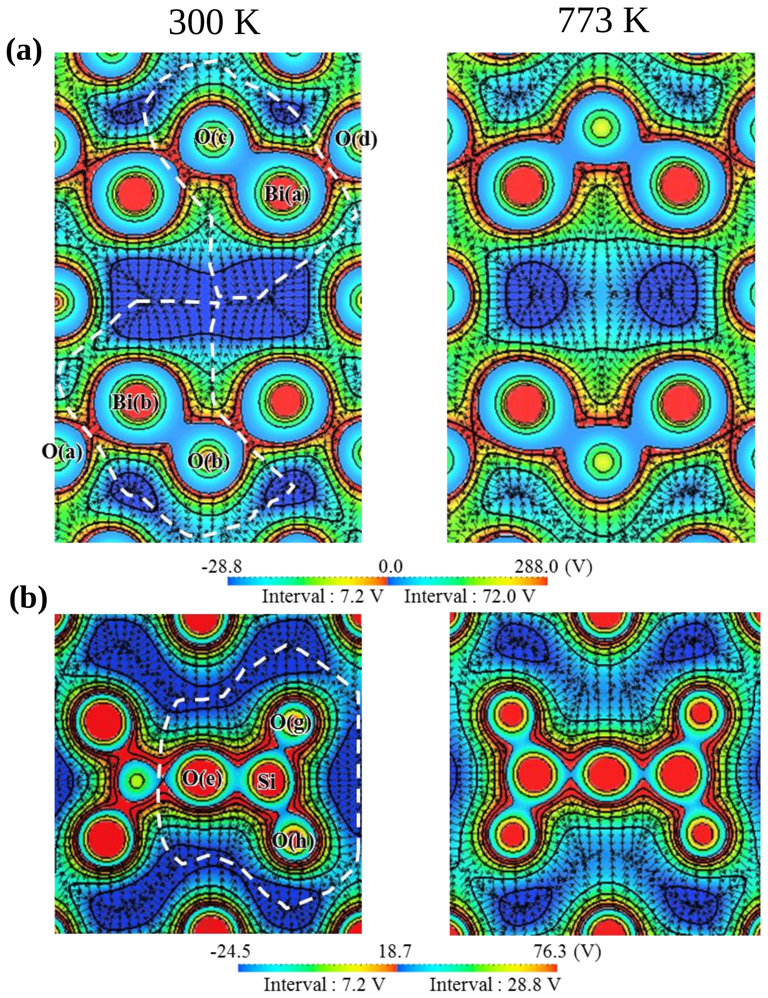

Figure S5 Two-dimensional electrostatic potential map of (a) the Bi_2O_2 and (b) the SiO_3 layer. For the Bi_2O_2 layer (SiO_3 layer), it was colored by the value of electrostatic potential from -28.8 V to 288.0 V (from -24.5 V to 76.3 V). Equipotential lines are drawn from -28.8 V to 0.0 V with 7.2 V intervals (from -24.5 V to 18.7 V with 7.2 V intervals) in low EP region and from 0.0 V to 288.0 V with 72.0 V intervals (from 18.7 V to 76.3 V with 28.8 V intervals) in high EP region, respectively. The black arrows are electric vectors calculated by the minus gradient of the electrostatic potential at each point.

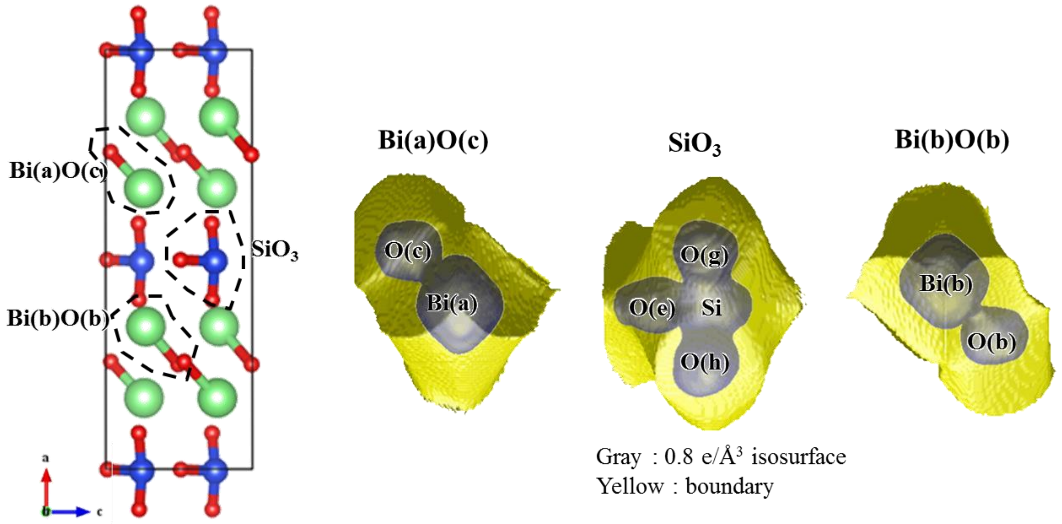

Figure S6 Fragment units determined by the local minimum value of electrostatic potential.

References

Brian, H. T. (2006). *Powder Diffr*. **21**, 67.

Fujiwara, A., Sugimoto, K., Shih, C. H., Tanaka, H., Tang, J., Tanabe, Y., Xu, J., Heguri, S., Tanigaki,
K. & Takata, M. (2012). *Phys. Rev. B.* **84**, 144305.

Kim, J., Fujiwara, A., Sawada, T., Kim, Y., Sugimoto, K., Kato, K., Tanaka, H., Ishikado, H., Shamoto, S. & Takata, M. (2013). *Submitted.*

Kim, J., Kato, K., Takata, M., Shibata, T. & Moritomo, Y. (2009). *Phys. Rev. B* **79**, 132105.

Kim, J., Tanaka, H., Kato, K., Takata, M. & Moritomo, Y. (2011). *Jpn. J. Appl. Phys.* **4**, 025801.

Sakata, M. & Sato, M. (1990). *Acta Crystallogr. A* **46**, 263-270.

Takata, M. & Sakata, M. (1996). *Acta Crystallogr. A* **52**, 287-290.

Takata, M. (2008). *Acta Crystallogr. A* **64**, 232-245.

Tanaka, H., Kuroiwa, Y. & Takata, M. (2006). *Phys. Rev. B* **74**, 172105.
